# Cecal Volvulus: An Uncommon Diagnosis in a Child with Down’s Syndrome

**DOI:** 10.34172/aim.2023.18

**Published:** 2023-02-01

**Authors:** Mohamed Zouari, Hana Ben Ameur, Nesrine Ben Saad, Najoua Kraiem, Wiem Rhaiem, Riadh Mhiri

**Affiliations:** ^1^Department of Pediatric Surgery, Hedi-Chaker Hospital, Sfax, Tunisia; ^2^Research Laboratory “Developmental and Induced Diseases” (LR19ES12), Faculty of Medicine of Sfax, University of Sfax, Sfax, Tunisia

**Figure 1:**
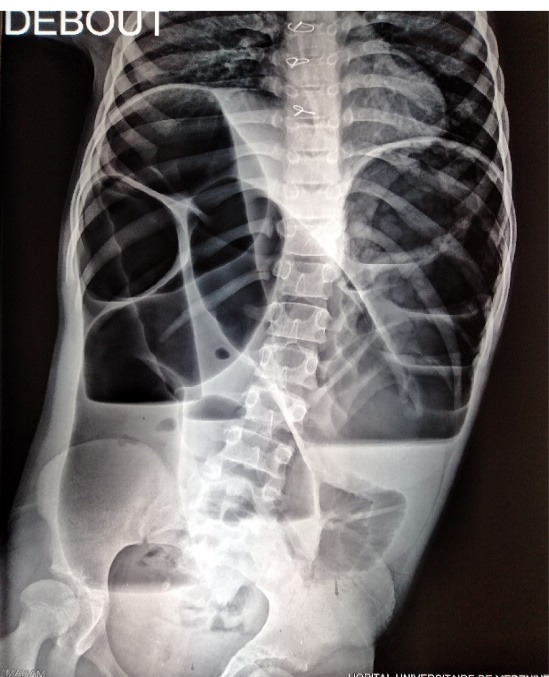
Plain Abdominal X-ray Showing Large Dilated Loops of Colonic Bowel Occupying the Majority of Abdominal Cavity with Multiple Air-fluid Levels.

**Figure 2:**
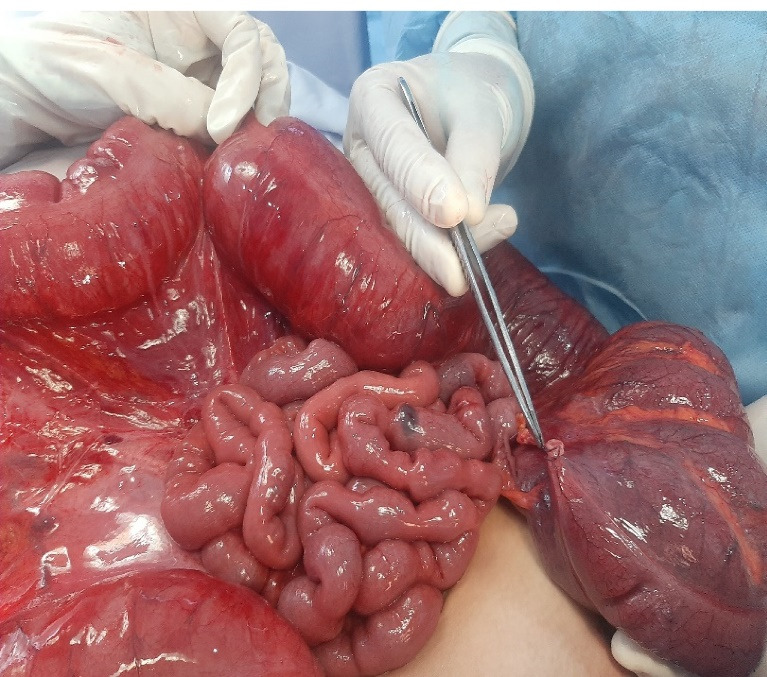
Huge Dilated Colon. After Reduction of Cecal Volvulus, the Cecum and the Ascending Colon were Viable.

 A 12-year-old female child with mental retardation and Down’s syndrome presented to the emergency department for abdominal distension and bilious vomiting of a 3-day duration. No stool was passed in the last 24 hours. The patient had no history of abdominal surgery and no similar episodes. However, she had a long history of constipation.

 Clinical examination revealed normal vital signs with a hugely distended tender abdomen. Rectal examination revealed stool but no blood. Radiography of the abdomen showed large dilated loops of colonic bowel occupying the majority of abdominal cavity with multiple air-fluid levels (Figure 1).

 At laparotomy, we identified a 270-degree counter-clockwise volvulus involving the cecum and ascending colon. There was no fixation to the lateral retroperitoneum. There were no signs of intestinal ischemia (Figure 2). Therefore, we performed cecal detorsion, cecopexy and appendectomy. The postoperative course was complicated by an enterocutaneous fistula. This fistula was successfully managed by non-surgical treatment including sepsis control, optimization of nutritional status, and wound care. We discharged the patient on day thirty-two after surgery. The child was followed as an outpatient for 4 years, and currently has not experienced any recurrence.

 Volvulus of the cecum is a diagnostic and therapeutic emergency. This condition, rare in adults, is extremely rare in children and accounts for less than 1% of acute intestinal obstructions in children.^1,2^ Several factors have been identified as risk factors for cecal volvulus, including malrotation, upward displacement of the cecum, colonic distension, and adhesions.^2^

 Unless managed in a timely fashion, strangulation, resulting from the twisting of the intestine around its mesenteric axis, will lead to intestinal ischemia and ultimately to gangrene or bowel perforation. Therefore, intestinal resection is necessary in 19% to 50% of cecal volvulus cases.

 Management of cecal volvulus is even more challenging in children with mental disability. These children commonly have aerophagia and constipation, which lead to bowel distension.^3^ The clinical presentation of cecal volvulus is usually non-specific. Therefore, a high suspicion level is essential to avoid diagnostic and therapeutic delays.^4^

 Management of cecal volvulus is still controversial. Several publications have reported the safety and efficacy of conservative management based on detorsion with or without cecopexy in the absence of intestinal ischemia. However, most authors strongly recommend surgical cecal resection, as the gold standard to treat this condition.^5-9^

## References

[1] Rosenblat JM, Rozenblit AM, Wolf EL, DuBrow RA, Den EI, Levsky JM (2010). Findings of cecal volvulus at CT. Radiology.

[2] Rakinic J. Colonic volvulus. In: Beck DE, Roberts PL, Saclarides TJ, Senagore AJ, Stamos MJ, Wexner SD, eds. The ASCRS Textbook of Colon and Rectal Surgery. New York, NY: Springer; 2011. p. 395-406. 10.1007/978-1-4419-1584-9_23.

[3] Takada K, Hamada Y, Sato M, Fujii Y, Teraguchi M, Kaneko K (2007). Cecal volvulus in children with mental disability. Pediatr Surg Int.

[4] Solis Rojas C, Vidrio Duarte R, García Vivanco DM, Montalvo-Javé EE (2020). Cecal volvulus: a rare cause of intestinal obstruction. Case Rep Gastroenterol.

[5] Mansoor K, Al Hamidi S, Khan AM, Samujh R (2009). Rare case of pediatric cecal volvulus. J Indian Assoc Pediatr Surg.

[6] Miura da Costa K, Saxena AK (2018). A systematic review of the management and outcomes of cecal and appendiceal volvulus in children. Acta Paediatr.

[7] Folaranmi SE, Cho A, Tareen F, Morabito A, Rakoczy G, Cserni T (2012). Proximal large bowel volvulus in children: 6 new cases and review of the literature. J Pediatr Surg.

[8] Madiba TE, Thomson SR (2002). The management of cecal volvulus. Dis Colon Rectum.

[9] van de Lagemaat M, Blink M, Bakx R, de Meij TG (2018). Cecal volvulus in children: is there place for colonoscopic decompression?. J Pediatr Gastroenterol Nutr.

